# Children’s eating attitudes test (ChEAT): validation and reliability in Turkish children

**DOI:** 10.1186/s40337-023-00811-1

**Published:** 2023-08-31

**Authors:** Osman Bozkurt, Betul Kocaadam-Bozkurt, Eda Köksal, Funda Seher Özalp Ateş

**Affiliations:** 1 Department of Nutrition and Dietetics, Faculty of Health Sciences, Erzurum Technical University, Erzurum, Turkey; 2 Department of Nutrition and Dietetics, Gazi University Faculty of Health Sciences, Ankara, Turkey; 3 Department of Biostatistics and Medical Informatics, Faculty of Medicine, Manisa Celal Bayar University, 45030 Manisa, Turkey

**Keywords:** Children’s eating attitudes test (ChEAT), Validation, Reliability, Turkish children

## Abstract

**Objectives:**

This study aims to examine the validity and reliability of the children’s eating attitudes test (ChEAT) in Turkish children.

**Methods:**

The participants were 331 children (137 boys and 194 girls, ages 8–15). Data was collected through face-to-face interviews using a questionnaire containing socio-demographic characteristics, the ChEAT, and the children's eating behaviour questionnaire (CEBQ). Exploratory factor analysis (EFA) was performed to determine the factor structure of the Turkish version of the ChEAT. Additionally, the reliability was examined in terms of internal consistency and test–retest reliability. All statistical analyses were performed using Mplus Trial Version and SPSS 11.5 (SPSS, Chicago, IL, USA).

**Results:**

According to the goodness-of-fit statistic, a three-factor solution was appropriate and compatible with clinical considerations. The three factors explained 50.1% of the variance. Cronbach’s alpha coefficient was 0.75 for ChEAT-26, 0.67 for “Preoccupation with thinness and food”, 0.63 for “Social pressure to eat”, and 0.71 for “Dieting”. Furthermore, the test–retest reliability was 0.72, 0.62, 0.59, and 0.59 respectively. Statistically significant correlations between the ChEAT and CEBQ were found (*p* < 0.05). “Preoccupation with thinness and food” was significantly higher in obese children (*p* < 0.05), while “Social pressure to eat” was lower (*p* < 0.001). Sex, grade, BMI, parental education in addition to working status affected the ChEAT-26 scores.

**Conclusions:**

The present study has provided preliminary evidence for the validity and reliability of a Turkish version of the ChEAT.

## Abstract (plain language summary)

Early diagnosis and intervention of abnormal eating attitudes are crucial to minimizing adverse physical as well as psychological health outcomes such as obesity or being underweight for children with eating disorders. Since eating disorders occur early, screening for nutritional attitudes along with behaviors is crucial. The Children's Eating Attitudes Test (ChEAT) was developed by Maloney et al. to evaluate eating attitudes and behaviors in children. This study aimed to adapt the ChEAT-26 to Turkish and evaluate its validity and reliability. The present study has provided preliminary evidence for the validity and reliability of a Turkish version of the ChEAT. High scores on ChEAT-26 indicate the severity of the eating disorder.

## Introduction

Obesity and eating disorders (EDs) adversely affect physical and mental health. These two significant public health issues are also connected [1, 2]. EDs are severe psychosomatic disorders common in adolescent girls. However, they can occur regardless of gender or age [3, 4]. Anorexia nervosa (AN), bulimia nervosa (BN), and binge eating disorder (BED)) are the most commonly studied EDs [5]. AN incidence begins to increase at around age 10. However, it is also reported in children aged 7–8 [6]. In a Canadian study, it was determined that between the ages of 5 and 12, early-onset EDs were found in 2.6 (95% confidence interval [CI], 2.1–3.2) cases per 100 000 person-years. Also, 47.1% of girls and 54.5% of boys showed signs of growth delay, and 46% of children were below the 10th percentile for body mass index [7]. In a recent study, among children in the United States, the prevalence of subclinical AN, BN, and BED was 6.0%, 0.2%, and 0.5%, respectively [8]. EDs in childhood and adolescence are correlated with overweight/obesity, reduced body esteem, and poor mental health [9, 10]. Considering that EDs occur early, it is crucial to screen for nutritional attitudes and behaviors which may lead to malnutrition [11].

Garner and Garfinkle [12] developed the 40-item Eating Attitudes Test (EAT) with the aim to evaluate adult eating attitudes and behaviors [12]. It was shown that the reliability and validity of the factor analysis remained unchanged when the number of items was reduced to 26. The EAT-26 is widely used worldwide to screen and evaluate symptoms along with the features of EDs [13]. Maloney et al. modified the EAT-26 to develop the Children's Eating Attitudes Test (ChEAT) in order to make it easier for children to grasp [14]. Therefore, some words were simplified, e.g., “terrified" was changed to “scared”, and “preoccupied with" was reworded as “think a lot about” [14]. The Children's Eating Behavior Questionnaire (CEBQ) was developed by Wardle et al. [15], a parent-report measure designed to assess variations in children's eating behaviors. Yilmaz et al. [16] conducted a Turkish reliability and validity study for CEBQ. While the CEBQ is a scale that evaluates eating behaviors reported primarily by parents, ChEAT is important because it is a self-administered scale by children used to evaluate EDs.

The ChEAT scale has been adapted in various countries, and its validity and reliability have been examined [11, 17-19]. The original EAT had three subscales: “Dieting, “Bulimia and Food Preoccupation”, and “Oral Control”. [13], while the ChEAT had no subscales in [14]. However, different factor structures were found in the validity and reliability studies of ChEAT in other languages. Additionally, some items were deleted from the scale [11, 17]. In Finnish children, the ChEAT was determined to have four factors with 24 items, and Cronbach's alpha value of the scale was found to be 0.84 [17]. Recently, in a Japanese study, the scale was determined as five factors with 25 items. Cronbach's alpha varies between 0.58 and 0.82 for subscales and 0.81 for the total scale [11]. A similar study in Spain revealed five factors with 26 items (Cronbach's alpha coefficient between 0.58 and 0.84 for subscales and 0.86 for the total scale) [19]. The different factor structures may be due to cultural differences. A similar structure may not be provided in every society and culture. Therefore, psychometric analyzes of ChEAT in Turkish culture are very important.

The prevalence of childhood and adolescent obesity is rising globally as well as in Turkiye [20]. The WHO European Regional Obesity Report 2022 has revealed that Turkiye has a higher obesity rate (32.1%) compared to that of other European nations (23.3%). On the other hand, no large sample-size studies evaluate the prevalence of eating disorders in children or adolescents in Turkiye [21]. Recent literature on the burden of eating disorders suggested that in individuals with eating disorders, quality of life is reduced, yearly healthcare expenditures are 48% greater than that in the general population, and mental health comorbidity is associated with 48% lower annual earnings [22]. Early diagnosis in addition to intervention of abnormal eating attitudes are crucial to minimizing adverse outcomes, which creates a need for a psychometrically reliable assessment tool. The ChEAT is the most widely used standardized self-report measure of symptoms and concerns characteristic of eating disorders worldwide [23]. This study aims to examine the validity and reliability of the ChEAT in Turkish children.

## Methods

### Participants

The study was carried out in elementary, middle, and high schools in Erzurum, Turkiye. Inclusion criteria include children aged 8–15 with no chronic or mental disorders. In order to conduct factor analysis, it is specified that the sampling must be taken at least five and preferably ten times the scale items number [24]. Our goal was to conduct 260 participants as there are 26 items in ChEAT. In total, 331 children participated in the study in case extreme and missing values would emerge. This study comprised 194 (58.6%) girls and 137 (41.4%) boys. The mean age of the children was 11.73 ± 2.19 years. In order to assess test–retest reliability, a subset of 93 children was invited to recomplete the ChEAT after 15 days.

### Procedure

Maloney et al., who developed the scale, were contacted via email for permission to adapt the Children's Eating Attitude Test into Turkish and exemine its reliability and validity in Turkish children [14]. Simple random selection was used to recruit participants from randomly selected schools in Erzurum (one of the metropolitan cities of Turkiye). The data was collected utilizing surveys and in-person interviews. The children along with their parents received two surveys (one child and one parent). Uncompleted surveys were not included.

Following the standard procedure recommended by Brislin [25] and Prieto [26], the scale was translated from English to Turkish by researchers proficient in both English and Turkish. Five academics proficient in English as well as Turkish in the field of nutrition and dietetics in addition to eating attitudes and behavior in children translated the original scale into Turkish. They contributed their opinions regarding the intelligibility of the scale and its relevance to Turkish culture. Following receipt of expert comments, all of the scale's items were revised. Then, the Turkish form of the scale was translated to English by five other Turkish academics specializing in nutrition and dietetics along with eating attitudes and behavior in children who had never seen the English form of the scale before and knew both languages and cultures. A professional translator was then consulted for approval on the Turkish and English translations of the ChEAT. Following consultation, the Turkish version was a close translation of the original version. The pre-application of the scale was undertaken with 30 children to assess the intelligibility of the questionnaire. In line with the opinions of the researchers, the ChEAT's items were easily comprehensible to children (see Appendix for Turkish translation).

Ethical permission was obtained from the Erzurum Technical University Ethics Committee (Meeting Number: 8; Decision Number: 4; and 29.08.2022) in addition to the Erzurum Provincial Directorate of National Education (19.09.2022). The study was carried out following the principles outlined in the Helsinki Declaration. Written informed consent was obtained from parents and verbal consent of the children was obtained.

### Measures

The survey for children contained general information, the Children's Eating Attitudes Test (ChEAT) [14], and anthropometric measurements. The children themselves responded to this survey. The survey of the parents contained general information and the Children's Eating Behavior Questionnaire (CEBQ) [16]. One of the parents (mother/father/guardian) responded to this questionnaire with questions regarding their children.

#### The children's eating attitudes test

Maloney et al. [14] modified the EAT-26 to develop the Children's Eating Attitudes Test-26 (ChEAT-26) with the aim to make it easier for children (8–15 years old) to grasp [14]. The ChEAT-26 self-administered questionnaire assesses dieting behavior and eating attitudes of children [27]. It consists of 26 items which are scored on a six-point Likert scale with the categories “never”, “rarely”, “sometimes”, “often”, “usually”, and “always”. The score is calculated by recording “never”, “rarely”, and “sometimes” categories as zero, “often” as one, “usually” as two, and “always” as three. Items 19 and 25 were reverse-coded, as suggested by other researchers [11, 17, 27]. The total original score ranges from 0 to 78. Higher scores indicate the severity of the eating disorder. Cronbach’s alpha coefficient was 0.76 in [14]. This study utilized the Turkish translation of the original “The Children’s Eating Attitudes Test”.

#### The children’s eating behavior questionnaire

The Turkish Children's Eating Behavior Questionnaire (CEBQ) consists of 35 Likert-type items assessed on a 5-point Likert scale by parents (1 = never, 5 = always). In the original study [15], in which the scale was developed, an eight-subscale factor structure formed during the scale's development. The eight subscale Cronbach alpha coefficients ranged from 0.74 to 0.91. The subscales are as follows: Food responsiveness, Enjoyment of food, Emotional overeating, Emotional undereating, Desire to drink, Slowness in eating, Satiety responsiveness, and Fussiness. Yilmaz et al. [16] adapted the Turkish version of this scale. Cronbach alpha coefficients for the Turkish version varied between 0.61 and 0.84 [16]. Each subscale is assessed independently. This scale was utilized to determine the external construct validity of the Turkish version of the ChEAT, as it evaluates eating behavior in children.

#### Anthropometric measurements

The body weight and height of the parents were taken based on the self-reports. The researchers carried out measurements in children. The height and weight of children were measured using the methods given by Lohman et al. [28]. According to age, weight, height, BMI, and Z-scores were calculated according to age, using the World Health Organization's growth standards [29] with use of the WHO AnthroPlus software (version 1.0.4, February 2011). The BMI of children was categorized according to the Z-score junctions [29].

### Statistical analysis

Exploratory factor analysis (EFA) with varimax rotation was performed to determine the factor structure of the Turkish version of the ChEAT-26. The number of factors was established based on the Scree plot and clinical considerations of factor structures. Exploratory factor analysis (EFA) for categorical data was applied using the mean and variance-adjusted weighted least squares (WLSMV) estimator which is an alternative method for ordinal data, in particular, which is not distributed normally, highly skewed or kurtic, or both [30]. Items with factor loadings above 0.30 were examined as salient. The associations between the factors of the Turkish version of ChEAT-26 and the factors of the Turkish Children’s Eating Behavior Questionnaire were assessed with Spearman Correlation Coefficient regarding external validity. Reliability was evaluated in terms of internal consistency and test–retest reliability. Cronbach’s alpha coefficient tested internal consistency [31]. Test–retest reliability was evaluated using the Intraclass Correlation Coefficient (ICC). A Cronbach alpha coefficient of < 0.39 indicates that it is not reliable, a value of 0.40–0.59 indicates low reliability, a value of 0.60–0.79 indicates that it is very reliable, and a value between 0.80 and 1.00 indicates that it is highly reliable [32].

Following determination of the factor structure, a group comparison was conducted using the subtotal scores for each factor based on EFA. A Mann–Whitney U Test and a Kruskal–Wallis variance analysis were used to compare the subscale scores regarding gender, class, parental education and working status as well as BMI-Z-score classification.

The post-hoc test for Kruskal–Wallis variance analysis was used to perform pairwise comparisons. Median (min.-max.) was used as descriptive statistics. All statistical analyses were performed using Mplus Trial Version and SPSS 11.5 (SPSS, Chicago, IL, USA).

## Results

### ChEAT-26 factor analysis

We initially performed EFA with varimax rotation assuming three, four, and five-factor solutions. According to the goodness-of-fit statistic, a three-factor solution was appropriate and compatible with clinical consideration. The Kaiser-Meyer Olkin (KMO) coefficient was 0.825; the Bartlett X^2^ was 2085.576, and *p* < 0.001. Items and factor loadings are given in Table 1. All 26 items loaded 0.30 or higher. The three factors were labeled as “Preoccupation with thinness and food”, “Social pressure to eat”, and “Dieting” (explained 50.1% of the total variance).

**Table 1:** Items and factor loadings of the items in the Turkish version of ChEAT-26

Items	Preoccupation with thinness and food (F1)	Social pressure to eating (F2)	Dieting (F3)
1. I am scared about being overweight	0.350		
3. I think about food a lot of time	0.582		
4. I have gone on eating binges where I feel that I might not be able to stop	0.837		
10. I feel very guilty after eating	0.594		
11. I think a lot about wanting to be thinner	0.622		
14. I think a lot about having fat on my body	0.711		
18. I think that food controls my life	0.427		
21. I give too much time and thought to food	0.644		
24. I like my stomach to be empty	0.482		
25. I enjoy trying new rich food	0.356		
8. I feel that others would like me to eat more		0.734	
13. Other people think I am too thin		0.723	
15. I take longer than others to eat my meals		0.379	
20. I feel that others pressure me to eat		0.640	
2. I stay away from eating when I am hungry			0.530
5. I cut my food into small pieces			0.300
6. I am aware of the energy (calorie) content in foods that I eat			0.586
7. I try to stay away from foods such as breads, potatoes, and rice			0.583
9. I vomit after I have eaten			0.550
12. I think about burning up energy (calories) when I exercise			0.300
16. I stay away from foods with sugar in them			0.696
17. I eat diet foods			0.639
19. I can show self-control around food			0.444
22. I feel uncomfortable after eating sweets			0.597
23. I have been dieting			0.668
26. I have the urge to vomit after eating			0.472
Eigenvalue	4.232	2.265	3.015
Explained variance (%)	23.17	12.71	14.22

In assessing the associations between subscales of the Turkish version of ChEAT-26 and subscales of the Turkish Children's Eating Behavior Questionnaire, statistically significant weak correlations were found between “Preoccupation with thinness and food”, and “Food responsiveness”, “Emotional overeating”, “Enjoyment of food”, “Desire to drink”, “Fussiness” (*p* < 0.05). There were statistically significant weak correlations between “Social pressure to eat” and “Enjoyment of food”, “Satiety responsiveness”, and “Slowness in eating”. In addition, there were statistically significant weak correlations between the 'ChEAT-26 score' and Satiety responsiveness, Slowness in eating, and Fussiness. However, no correlation was found between the “Dieting factor” of the Turkish version of ChEAT-26 or any factor of the Turkish Children's Eating Behavior Questionnaire (Table 2).

**Table 2:** Correlation Coefficients between factors of the Turkish version of ChEAT-26 and factors of the Turkish children’s eating behavior questionnaire

	Preoccupation with thinness and food (F1)		Social pressure to eating (F2)		Dieting (F3)		Total score	
	r	*p*	r	*p*	r	*p*	r	*p*
Food responsiveness	− 0.207	** < 0.001**	− 0.082	0.136	− 0.061	0.267	0.057	0.300
Emotional overeating	− 0.218	** < 0.001**	− 0.094	0.088	− 0.066	0.231	0.057	0.300
Enjoyment of food	− 0.184	**0.001**	− 0.297	** < 0.001**	0.009	0.876	− 0.002	0.973
Desire to drink	− 0.123	**0.026**	0.081	0.142	0.032	0.568	0.101	0.067
Satiety responsiveness	0.015	0.791	0.260	** < 0.001**	0.026	0.640	0.113	**0.041**
Slowness in eating	− 0.024	0.668	0.287	** < 0.001**	0.086	0.120	0.138	**0.012**
Emotional undereating	0.059	0.281	0.102	0.065	0.059	0.288	0.075	0.172
Fussiness	0.226	** < 0.001**	− 0.103	0.061	0.082	0.139	0.114	**0.039**

The bold values are indicates significant at *p* < 0.05 or *p* < 0.001

### Reliability

Cronbach’s alpha coefficient was 0.75 for ChEAT-26, 0.67 for “Preoccupation with thinness and food”, 0.63 for “Social pressure to eat”, and 0.71 for “Dieting”. The test–retest reliability ICC (%95 Confidence Interval) was 0.72 (0.63–0.79) for ChEAT-26, 0.62 (0.59–0.70) for Preoccupation with thinness and food, 0.59 (0.57–0.71) for Social pressure to eat and 0.59 (0.55–0.69) for Dieting.

### Group differences

The median (min–max) ChEAT-26 score was 12.0 (0.0–51.0) in the study. When evaluated according to gender, it was determined as 13.0 (0.0–44.0) in girls and 11.0 (0.0–51.0) in boys (*p* < 0.05) (Table 3). The scores for ChEAT-26 and “Social pressure to eat" were higher for girls (*p* < 0.05). The “Dieting" factor scores for the class examination were statistically significant (*p* = 0.008). Post-hoc tests showed differences between the 3rd–5th, 4th–5th, 3rd–8th, and 4th–8th years. In addition, the ‘Preoccupation with thinness and food’ score in mothers with ≤ 8 years of education and the 'Social pressure to eat' score in mothers who were unemployed were significantly higher (*p* < 0.05). When the BMI-for-age Z score (BAZ) classification of children was considered, “Preoccupation with thinness and food” was significantly higher in obese children (*p* < 0.05), while “Social pressure to eat” was lower (*p* < 0.001) (Table 4).

**Table 3:** Medians (min–max) of ChEAT and its subscale scores by demographic classification

	n (%)	Total score	*p*-value	Preoccupation with thinness and food (F1)	*p*-value	Social pressure to eating (F2)	*p*-value	Dieting (F3)	*p*-value
*Gender*									
Boys	137 (41.4)	11.0 (0.0–51.0)	**0.024**	3.0 (0.0–18.0)	**0.019**	1.0 (0.0–14.0)	0.243	4.0 (0.0–24.0)	0.185
Girls	194 (58.6)	13.0 (0.0–44.0)		4.0 (0.0–27.0)		2.0 (0.0–15.0)		5.0 (0.0–24.0)	
*Class*									
3rd	30 (9.1)	12.5 (3.0–34.0)	0.277	3.0 (0.0–12.0)	0.256	2.5 (0.0–14.0)	0.652	3.0 (0.0–22.0)	**0.008**
4th	122 (36.9)	14.0 (0.0–51.0)		4.0 (0.0–18.0)		2.0 (0.0–14.0)		3.0 (0.0–9.0)	
5th	30 (9.1)	7.0 (0.0–29.0)		2.5 (0.0–13.0)		1.5 (0.0–10.0)		2.5 (0.0–15.0)	
6th	20 (6.0)	12.0 (0.0–20.0)		4.0 (0.0–12.0)		2.5 (0.0–9.0)		4.0 (0.0–8.0)	
7th	30 (9.1)	13.0 (1.0–33.0)		4.0 (0.0–27.0)		1.5 (0.0–10.0)		4.0 (0.0–16.0)	
8th	29 (8.8)	13.0 (0.0–40.0)		7.0 (0.0–22.0)		2.0 (0.0–12.0)		5.5 (0.0–24.0)	
9th	70 (21.1)	11.0 (0.0–39.0)		3.5 (0.0–15.0)		1.5 (0.0–15.0)		7.0 (0.0–24.0)	
*Years of education of the mother*									
≤ 8 years	156 (47.1)	13.0 (0.0–40.0)	0.253	5.0 (0.0–22.0)	**0.037**	2.0 (0.0–14.0)	0.835	4.0 (0.0–24.0)	0.926
> 8 years	175 (52.9)	11.0 (0.0–51.0)		3.0 (0.0–27.0)		2.0 (0.0–15.0)		4.0 (0.0–24.0)	
*Years of education of the father*									
≤ 8 years	109 (32.9)	12.0 (0.0–36.0)	0.282	4.0 (0.0–16.0)	0.109	2.0 (0.0–14.0)	0.399	4.0 (0.0–15.0)	0.472
> 8 years	222 (67.1)	12.0 (0.0–51.0)		3.0 (0.0–27.0)		2.0 (0.0–15.0)		4.0 (0.0–24.0)	
*Mother’s working status*									
Working	81 (24.5)	10.0 (1.0–44.0)	0.147	3.0 (0.0–27.0)	0.508	1.0 (0.0–14.0)	**0.040**	4.0 (0.0–24.0)	0.590
Not working	250 (75.5)	13.0 (0.0–51.0)		4.0 (0.0–22.0)		2.0 (0.0–15.0)		4.0 (0.0–24.0)	
*Father’s working status*									
Working	317 (%95.8)	12.0 (0.0–51.0)	0.273	3.0 (0.0–27.0)	0.312	2.0 (0.0–15.0)	0.551	4.0 (0.0–24.0)	0.181
Not working	14 (%4.2)	14.5 (1.0–30.0)		6.5 (0.0–13.0)		1.5 (0.0–10.0)		6.5 (0.0–17.0)	

The bold values are indicates significant at *p* < 0.05 or *p* < 0.001

**Table 4:** Medians (Min–Max) of ChEAT ant its subscale scores by children’s BAZ classification

	n (%)	ChEAT-26	*p*- value	Preoccupation with thinness and food (F1)	*p*-value	Social pressure to eating (F2)	*p*-value	Dieting (F3)	*p*-value
*BAZ*									
Underweight	41 (%12.4)	12.0 (1.0–33.0)	0.753	3.0 (0.0–15.0)^a^	**0.038**	3.0 (0.0–14.0)^a^	** < 0.001**	4.0 (0.0–14.0)	0.408
Normal	187 (%56.5)	12.0 (0.0–44.0)		4.0 (0.0–17.5)^a,b^		3.0 (0.0–15.0)^a^		4.0 (0.0–24.0)	
Overweight/obese	103 (%31.1)	12.0 (0.0–51.0)		5.0 (0.0–27.0)^b^		1.0 (0.0–11.0)^b^		4.0 (0.0–24.0)	

^a,^^b^The groups with the same letters within a column are not significantly different according to pairwise comparisons

The bold values are indicates significant at *p* < 0.05 or *p* < 0.001

## Discussion

This study aimed to adapt the Turkish version of the ChEAT-26 and evaluate its validity and reliability, an internationally recognized scale for assessing disordered eating attitudes, for a representative sample of Turkish elementary, middle, and high school students. The present study has provided preliminary evidence for the validity and reliability of a Turkish version of the ChEAT.

While the Japanese, Belarusian, and Spanish versions of the ChEAT had five factors [11, 18, 19], the Portuguese and Finnish versions had four factors [17, 33]. In this study, the goodness-of-fit statistics obtained from the three-factor structure were sufficient. This solution was also in line with clinical consideration. All 26 items loaded 0.30 or higher. The 'Social pressure to eat' subscale and its items were the same as that in the Japanese study [11]. The different factor structures may be due to cultural differences. A similar structure may not be provided in every society and culture. In our study, three factors with 26 items were appropriate and compatible with clinical consideration.

In our study, Cronbach's alpha coefficient was 0.75 for ChEAT-26, 0.67 for “Preoccupation with thinness and food”, 0.63 for “Social pressure to eat”, and 0.71 for “Dieting". These results show that the Turkish version of ChEAT has good internal consistency. In addition, our results revealed that the scale has a good test–retest reliability. In the validity and reliability study conducted with Finnish children, Cronbach's alpha value of the scale was found to be 0.84 [17]. Recently, in a Japanese study, Cronbach's alpha has been found to vary between 0.58 and 0.82 for subscales and 0.81 for the total scale [11]. In a similar study conducted in Spain, Cronbach's alpha coefficient was between 0.58 and 0.84 for subscales and 0.86 for the total scale [19]. Several researchers have investigated an appropriate age cut-off for children [11, 19]. In age groups between 9 and 17, cut-offs ranging from 10 to 20 have been examined. However, there is no consensus on the appropriate cut-off [11, 14, 19]. Due to the lack of eating disorder diagnoses, we were unable to investigate a ChEAT cut-off for recognizing eating disorder symptoms.

The ChEAT is the most widely used standardized self-report measure of symptoms and concerns characteristic of eating disorders worldwide [23]. While the CEBQ is a scale that evaluates eating behaviors reported primarily by parents, CheAT is important because it is a self-administered scale used to evaluate eating behavior disorders. This study examined correlations between ChEAT-26 and its subscales and CEBQ. Significant weak correlations were found. In particular, the Satiety responsiveness and Slowness in eating subscales of CEBQ may be closely related to EDs. In our study, it was determined that these subscales showed weak positive correlations with ChEAT scores. These results demonstrate that the Turkish version of the ChEAT-26 has moderate external construct validity with CEBQ.

The ChEAT-26 may enhance awareness of factors (sex, grade, socio-economic status, and parental education levels) which can influence eating attitudes in school children [19]. In this study, the ChEAT score was significantly higher in girls than in boys, indicating that the tendency in girls to have EDs may be higher than boys. Moreover, the fact that girls had higher Preoccupation with thinness and food scores than boys suggests that girls are more preoccupied with thinness than boys. These results are similar to previous research [19, 34]. A meta-analysis reported that females had more body dissatisfaction compared to males [35]. Furthermore, typically, females face greater pressure to achieve an ideal body shape [36]. Therefore, promoting healthy eating attitudes and habits in addition to examining distorted attitudes about thinness is essential.

In a Japanese study, the mean ChEAT score decreased with increasing grades [11]. According to Suzuki et al., the prevalence of EDs among junior high school children increased linearly and peaked in ninth grade [37]. Similar results were also found for the Dieting subscale in our study. Previous studies indicate increased EDs, especially in adolescence [38, 39]. Studies reveal that negative body image and EDs are related and affect adolescent health [40]. Media influence and peer interactions shape the body image of adolescents [36]. Body image is essential to adolescent health, and precautions should be taken to promote a healthy body image among adolescents to prevent EDs.

The “Preoccupation with thinness and food” score was significantly higher in the children compared to that of mothers whose education period was ≤ 8 years and the “Social pressure to eat” score was significantly higher in the children of mothers who were unemployed (*p* < 0.05). Families play a major role in the development of the eating behaviors of children. It should be emphasized that family members impact the eating habits of one another [41]. Literature indicates that parental education, the occupation of the mother along with parental and teacher health consciousness are positively associated with healthy eating behaviors in children [42]. The findings of our study also indicate that parental education and occupation may influence children's eating behaviors.

According to the BAZ classification of the children, Preoccupation with thinness and food subscale scores was significantly higher in obese children (*p* < 0.05). In contrast, Social pressure to eat scores were lower (*p* < 0.001). Additionally, ChEAT distinguishes between children with and with no overeating. Previous research indicates that overweight children are more prone to EDs [43, 44]. These results show that the risk of EDs may increase in obese children.

### Limitations and future research

This study had some limitations. Firstly, the inclusion criteria for this study was children aged 8–15 with no chronic or mental disorders, based on self-reports from parents. However, we did not conduct structured diagnostic and cognitive interviews with participants in this study. It is possible that children with eating disorders or other psychiatric disorders were included. Secondly, we had no ED diagnoses; we could not evaluate a ChEAT cut-off point for disordered eating symptoms. Since there is a need for studies on eating disorders in Turkish children, this scale in prevalence/clinical/cause-effect studies will significantly contribute to the literature.

## Conclusion

In conclusion, the present study has provided preliminary evidence for the validity and reliability of a Turkish version of the ChEAT. Factor analysis identified three factors. Due to no cut-off score, higher scores indicate the severity of the eating disorder. We propose using the Turkish version of ChEAT-26 to evaluate students for EDs in schools for epidemiological studies. Sex, grade, BMI, parental education, and working status affected the ChEAT-26 scores. Therefore, further research is needed to investigate the relationship between these factors and EDs in children.

## Data Availability

The datasets used during the present study can be obtained from the corresponding author on reasonable request.

## Appendix: Çocuklar için Yeme Tutum Testi-26

**Table:**

	Her zaman	Çoğunlukla	Sık Sık	Bazen	Nadiren	Hiçbir zaman
1. Fazla kilolu/şişman olmaktan korkarım						
2. Acıktığımda yemek yemekten kaçınırım						
3. Çoğu zaman yemeği düşünürüm						
4. Yemek yemeyi durduramadığım zamanlar olur						
5. Yiyeceklerimi küçük küçük parçalara bölerim						
6. Yediğim besinlerin enerji (kalori) içeriklerini bilirim						
7. Ekmek, pirinç, patates gibi yiyeceklerden uzak durmaya çalışırım						
8. Başkaları/çevremdekiler benim daha fazla yememi istiyor gibi gelir						
9. Yemek yedikten sonra kusarım						
10. Yemek yedikten sonra çok suçluluk duyarım						
11. Daha zayıf olmak konusunda çok düşünürüm						
12. Egzersiz yaparken harcadığım enerjiyi (kalorileri) düşünürüm						
13. Başkaları/çevremdekiler benim çok zayıf olduğumu düşünür						
14. Vücudumun yağlandığı konusunda çok düşünürüm						
15. Başkalarına/çevremdekilere göre yemek yemem daha uzun sürer						
16. Şekerli yiyeceklerden kaçınırım						
17. Diyet besinleri tüketirim						
18. Yiyeceklerin benim hayatımı kontrol ettiğini düşünürüm						
19. Yiyecekler konusunda kendimi denetleyebilirim						
20. Yemek yemem konusunda başkalarının bana baskı yaptığını hissederim						
21. Yemeklerle ilgili çok düşünürüm ve zaman harcarım						
22. Tatlı (şekerli besinler) yedikten sonra kendimi rahatsız hissederim						
23. Diyet yapıyorum						
24. Midemin boş olmasından hoşlanırım						
25. Yeni yüksek kalorili yiyecekleri denemekten hoşlanırım						
26. Yedikten sonra kusma dürtüsü hissederim						

## References

[1] Bray I, Slater A, Lewis-Smith H, Bird E, Sabey A (2018). Promoting positive body image and tackling overweight/obesity in children and adolescents: a combined health psychology and public health approach. Prev Med.

[2] Da Luz FQ, Sainsbury A, Mannan H, Touyz S, Mitchison D, Hay P (2017). Prevalence of obesity and comorbid eating disorder behaviors in South Australia from 1995 to 2015. Int J Obes.

[3] Fairburn CG, Harrison PJ (2003). Risk factors for anorexia nervosa. Lancet.

[4] Steinhausen HC, Villumsen MD, Hørder K, Winkler LAD, Bilenberg N, Støving RK (2021). Comorbid mental disorders during long-term course in a nationwide cohort of patients with anorexia nervosa. Int J Eat Disord.

[5] Mandera-Grygierzec A, Kostrzewska P, Szuster E, Pawlikowska-Gorzelańczyk A, Lebioda A (2022). Eating disorders in children and adolescents-the current state of knowledge. J Educ Health Sport.

[6] Baş M (2019). Adölesanlarda Yeme Bozuklukları. Turkiye klinikleri Nutr Dietetics Spec Top.

[7] Pinhas L, Morris A, Crosby RD, Katzman DK (2011). Incidence and age-specific presentation of restrictive eating disorders in children: a Canadian paediatric surveillance program study. Arch Pediatr Adolesc Med.

[8] Murray SB, Ganson KT, Chu J, Jann K, Nagata JM (2022). The prevalence of preadolescent eating disorders in the United States. J Adolesc Health.

[9] Convertino AD, Blashill AJ (2022). Psychiatric comorbidity of eating disorders in children between the ages of 9 and 10. J Child Psychol Psychiatry.

[10] Wade KH, Kramer MS, Oken E, Timpson NJ, Skugarevsky O, Patel R, Martin RM (2017). Prospective associations between problematic eating attitudes in midchildhood and the future onset of adolescent obesity and high blood pressure. Am J Clin Nutr.

[11] Chiba H, Nagamitsu S, Sakurai R, Mukai T, Shintou H, Koyanagi K, Matsuishi T (2016). Children's eating attitudes test: reliability and validation in Japanese adolescents. Eat Behav.

[12] Garner DM, Garfinkel PE (1979). The eating attitudes test: an index of the symptoms of anorexia nervosa. Psychol Med.

[13] Garner DM, Olmsted MP, Bohr Y, Garfinkel PA (1982). The eating attitudes test: psychometric features and clinical correlations. Psychol Med.

[14] Maloney MJ, McGUIRE JB, Daniels SR (1988). Reliability testing of a children's version of the eating attitude test. J Am Acad Child Adolesc Psychiatry.

[15] Wardle J, Guthrie CA, Sanderson S, Rapoport L (2001). Development of the children's eating behaviour questionnaire. J Child Psychol Psychiatry Allied Discipl.

[16] Yılmaz R, Esmeray H, Erkorkmaz Ü. Çocuklarda Yeme Davranışı Anketinin Türkçe uyarlama çalışması. Anatol J Psychiatry/Anadolu psikiyatri dergisi. 2011;12(4).

[17] Lommi S, Viljakainen HT, Weiderpass E, de Oliveira Figueiredo RA (2020). Children’s eating attitudes test (ChEAT): a validation study in Finnish children. Eating Weight Disord Stud Anorex Bulim Obes.

[18] Murphy TJ, Hwang H, Kramer MS, Martin RM, Oken E, Yang S (2019). Assessment of eating attitudes and dieting behaviors in healthy children: confirmatory factor analysis of the children's eating attitudes test. Int J Eat Disord.

[19] Rojo-Moreno L, García-Miralles I, Plumed J, Barberá M, Morales MM, Ruiz E, Livianos L (2011). Children's eating attitudes test: validation in a sample of Spanish schoolchildren. Int J Eat Disord.

[20] World Health Organization. Consideration of the evidence on childhood obesity for the Commission on Ending Childhood Obesity: report of the ad hoc working group on science and evidence for ending childhood obesity. World Health Organization Geneva, Switzerland. 2016.

[21] World Health Organization European Regional Obesity Report 2022. Copenhagen: WHO Regional Office for Europe; 2022. Licence: CC BY-NC-SA 3.0 IGO.

[22] van Hoeken D, Hoek HW (2020). Review of the burden of eating disorders: mortality, disability, costs, quality of life, and family burden. Curr Opin Psychiatry.

[23] Ayala CO, Scarpatto C, Garizábalo-Davila CM, Valencia PAD, Irigaray TQ, Cañon-Montañez W, Mattiello R (2022). Assessing eating disorder symptoms in low and middle-income countries: a systematic review of psychometric studies of commonly used instruments. J Eat Disord.

[24] Osborne JW, Costello AB (2004). Sample size and subject to item ratio in principal components analysis. Pract Assess Res Eval.

[25] Brislin RW, Lonner WL, Berry JW (1986). The wording and translation of research instruments. Cross-cultural research and methodology series field methods in cross-cultural research.

[26] Prieto AJ (1992). A method for translation of instruments to other languages. Adult Educ Q.

[27] Maloney MJ, McGuire J, Daniels SR, Specker B (1989). Dieting behavior and eating attitudes in children. Pediatrics.

[28] Lohman T, Roche A, Martorell R (1991). Human kinetics books, abridged; anthropometric standardization reference manual.

[29] Onis MD, Onyango AW, Borghi E, Siyam A, Nishida C, Siekmann J (2007). Development of a WHO growth reference for school-aged children and adolescents. Bull World Health Organ.

[30] Muthén BO, Bollen KA, Long JS (1993). Goodness of fit with categorical and other non-normal variables. Testing structural equation models.

[31] Gönüllü İ, Öztuna D. A Turkish adaptation of the student version of the Jefferson scale of physician empathy. Marmara Med J. 2012;25(2).

[32] Alpar R (2012). Spor, sağlık ve eğitim bilimlerinde örneklerle uygulamalı istatistik ve geçerlik güvenirlik.

[33] Teixeira MDCB, Pereira ATF, Saraiva JMT, Marques M, Soares MJ, Bos SC, Macedo AJFD (2012). Portuguese validation of the children's eating attitudes test. Arch Clin Psychiatry (São Paulo).

[34] Ferreiro F, Seoane G, Senra C (2011). A prospective study of risk factors for the development of depression and disordered eating in adolescents. J Clin Child Adolesc Psychol.

[35] Myers TA, Crowther JH (2009). Social comparison as a predictor of body dissatisfaction: a meta-analytic review. J Abnorm Psychol.

[36] Linardon J, McClure Z, Tylka TL, Fuller-Tyszkiewicz M (2022). Body appreciation and its psychological correlates: a systematic review and meta-analysis. Body Image.

[37] Suzuki H, Ohara T, Horikawa R, Ogawa Y (2013). The epidemiology survey of the anorexia nervosa by the questionnaire to teachers in charge of health education of high schools in Tokyo. Jpn J Psychosom Intern Med.

[38] Golden NH, Schneider M, Wood C, Daniels S, Abrams S, Corkins M, Braverman PK. Preventing obesity and eating disorders in adolescents. Pediatrics. 2016;138(3). 10.1542/peds.2016-1649 27550979

[39] Wu J, Lin Z, Liu Z, He H, Bai L, Lyu J (2022). Secular trends in the incidence of eating disorders in China from 1990 to 2017: a joinpoint and age–period–cohort analysis. Psychol Med.

[40] Hartman-Munick SM, Gordon AR, Guss C (2020). Adolescent body image: influencing factors and the clinician's role. Curr Opin Pediatr.

[41] Kitzman-Ulrich H, Wilson DK, St George SM, Lawman H, Segal M, Fairchild A (2010). The integration of a family systems approach for understanding youth obesity, physical activity, and dietary programs. Clin Child Fam Psychol Rev.

[42] Xu J (2022). The roles of family and school members in influencing children’s eating behaviours in china: a narrative review. Children.

[43] Lydecker JA, Rossa ET, Grilo CM (2022). Does your past define you? How weight histories are associated with child eating-disorder psychopathology. Eating Weight Disord Stud Anorex Bulim Obes.

[44] Matthews A, Kramer RA, Mitan L (2022). Eating disorder severity and psychological morbidity in adolescents with anorexia nervosa or atypical anorexia nervosa and premorbid overweight/obesity. Eating Weight Disord Stud Anorex Bulim Obes.

